# Right atrial isomerism diagnosed by STIC-HD live flow and autopsy: A case report

**DOI:** 10.1097/MD.0000000000024912

**Published:** 2021-02-26

**Authors:** Linhua Yang, Liuying Zhou, Lin Chen

**Affiliations:** Department of Ultrasound, Chengdu Women's and Children's Central Hospital, School of Medicine, University of Electronic Science and Technology, Chengdu, China.

**Keywords:** autopsy, diagnose, right atrial isomerism, STIC-HD live flow

## Abstract

**Rationale:**

Right atrial isomerism (RAI) is one of the most severe forms of congenital heart disease. This case of RAI was so complex that it incorporated 7 heart defects. It can be challenging to display the spatial relationship between different anatomical structures using conventional two-dimensional and color ultrasound (2D-Doppler imaging); therefore, we used spatio-temporal image correlation (STIC) and high definition live flow imaging technology to vividly display this case of RAI in a stereoscopic mode.

**Patient concerns:**

A 24-year-old woman was referred to our tertiary center at 24 weeks of gestation. The woman had difficult conceiving. Once pregnant, she was opposed to abortion, even if there was a possibility of deformity.

**Diagnosis:**

The fetus presented with an atrioventricular septal defect, persistent left superior vena cava, supra-cardiac total anomalous pulmonary venous connection (TAPVC), double outlet right ventricle, right ductus arteriosus, right aortic arch (RAA) with mirror image branching, and aortic arch dysplasia.

**Interventions:**

After consulting a pediatric cardiologist, the woman requested an abortion and consented to an autopsy.

**Outcomes:**

Autopsy supported the echocardiographic findings.

**Lessons:**

Accurate diagnosis of RAI is essential for clinical and parent decision making. 2D-Doppler imaging combined with STIC-HD live flow can be used to visualize the spatial morphology of blood vessels, including the cardiac chambers and great vessels of the fetal heart, and smaller peripheral vessels.

## 1 Introduction

Right atrial isomerism (RAI) is a subset of heterotaxy syndrome, in which some paired structures on opposite sides of the left-right axis of the body have the morphology of normal right-sided structures and are symmetrical mirror images of each other.^[1]^ RAI is one of the most severe forms of congenital heart disease. Patients with RAI present with a complete atrioventricular septal defect, total anomalous pulmonary venous return (TAPVC) and discordant ventriculo-arterial connections, as well as anomalies of other thoracic and abdominal organs such as asplenia, bilateral morphologically right atrial appendage, bilateral right-sided trilobed lungs, a right-sided stomach and a midline liver.^[2]^

It can be challenging to display the spatial relationship between different anatomical structures using conventional two-dimensional color Doppler ultrasound. In contrast, spatio-temporal image correlation (STIC) and high definition live flow imaging technology can vividly display the spatial relationships between blood vessels in a stereoscopic mode. Consequently, STIC-HD live flow can be used to visualize the spatial morphology of blood vessels, including the cardiac chambers and great vessels of the fetal heart, smaller peripheral vessels, and placenta blood vessels.^[3]^ Several reports have described the cardiac defects associated with RAI.^[4]^ Here, we used STIC-HD live flow to comprehensively investigate the complexity of the heart defects in a fetus presenting with RAI, which were confirmed by autopsy. Our findings should raise awareness of heterotaxy syndrome among clinicians and ultrasonographers.

## 2 Case presentation

A 24-year-old gravida 1, para 0 woman was referred to our tertiary center at 24 weeks of gestation because the fetal heart demonstrated a single ventricle and endocardial cushion defect. The spouse was healthy, and there was no family history of congenital heart disease. Fetal biometric measurements were consistent with gestational age. Screening for Down's syndrome at 17 weeks of gestation was negative. Chromosome microarray analysis performed on cells obtained through amniocentesis revealed no clinically significant cytogenetic information.

After providing written informed consent, the woman underwent detailed fetal echocardiography, which was performed with a GE 10 Ultrasound system (Tiefenbach, Austria) equipped with a 2- to 7-MHz convex probe (GE). Findings showed

**Figure 1:**
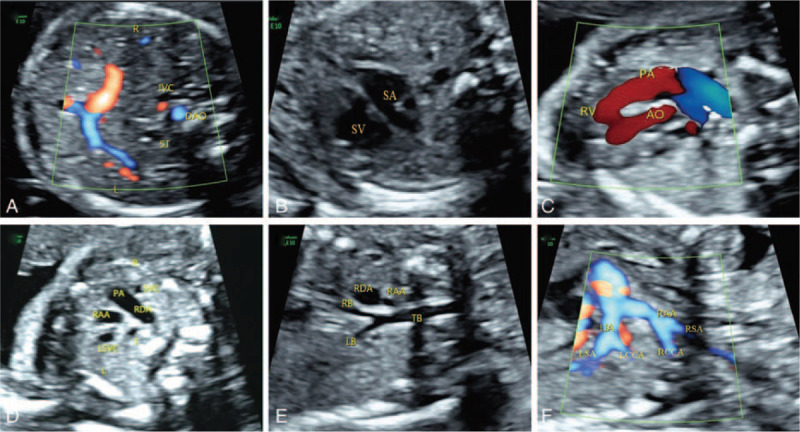
2D image. (A) IVC and descending AO on the right side. The stomach was near the midline; (B) Intracardiac anomalies included a single atrium, single ventricle and common atrioventricular valve; (C) Double outlet right ventricle; (D) Three vessel and trachea view showing the aortic arch and the ductus arteriosus were located on the right side of the trachea and a persistent left superior vena cava; (E) The aortic arch and ductus arteriosus on the right side of the trachea, aortic arch dysplasia; (F) The left innominate artery originated as the most proximal branch of the aortic arch, followed by the RCCA and RSA, and the LIA, branched LCCA and LSA. (DAO = descending aorta, IVC = inferior vena cava, LB = left principal bronchus, LCCA = left common carotid artery, LIA = left innominate artery, LSA = left subclavian artery, LSVC = left superior vena cava, PA = pulmonary artery, RAA = right aortic arch, RB = right principal bronchus, RCCA = right common carotid artery, RDA = right ductus arteriosus, RSA = right subclavian artery, RV = right ventricle, SA = single atrium, ST = stomach, SV = single ventricle, SVC = superior vena cava, T = trachea, TB = trachea bronchus).

**Figure 2:**
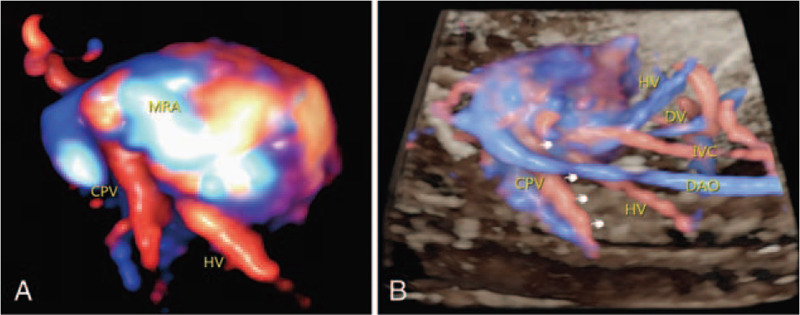
STIC-HD live flow (A) The PV drained into the CPVC, and the CPVC connected to the superior vena cava, indicating supra-cardiac TAPVC; (B) IVC and descending AO on the right side; the ductus venosus ran from the umbilical vein to the IVC, the hepatic vein drained into the IVC. (CPV = common pulmonary vein, DAO = descending aorta, DV = ductus venosus, HV = hepatic vein, IVC = inferior vena cava, MRA = morphological right atrium, PV = pulmonary vein).

1. a left-sided stomach, a midline liver, and a right-sided abdominal aorta and inferior vena cava on a transverse section of the fetal abdomen (Fig. 1A);
2. a single atrium, single ventricle and common atrioventricular valve on the 4 chamber view (Fig. 1 B);
3. a double outlet right ventricle on the left and right ventricular outflow tract view (Fig. 1C);
4. supra-cardiac TAPVC on the suprasternal view using STIC-HD live flow, in which the common pulmonary veins obviously drained via an anomalous vertical vein into the superior vena cava system (Fig. 2 A, B);
5. the left innominate artery originated as the most proximal branch of the right aortic arch, followed by the right common carotid artery and the right subclavian artery (RSA), and the LIA, branched left common carotid artery and left subclavian artery on the descending aortic arch coronary view (Fig. 1 F);
6. the ductus arteriosus and the RAA were on the right side of the trachea (Fig. 1 D, E), and there was a persistent left superior vena cava (Fig. 1 D) and aortic arch dysplasia (Fig. 1 E) on the 3 vessel and trachea view.

**Figure 3:**
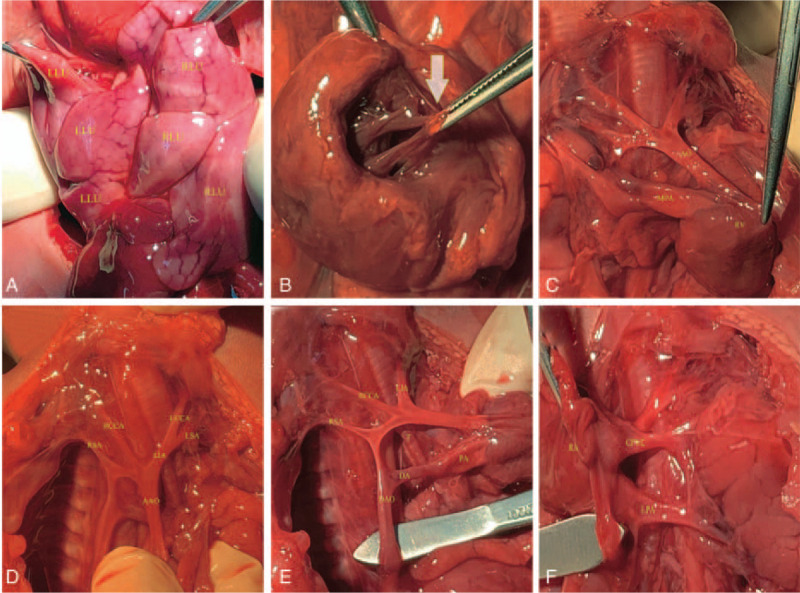
Autopsy findings. (A) Bilateral trilobed lung; (B) Common atrioventricular valve (arrow); (C) Double outlet right ventricle; (D) The aortic arch was located on the right side of the trachea. The left innominate artery originated as the most proximal branch of the aortic arch, followed by the RCCA and RSA, and the LIA, branched LCCA and LSA; (E) The ductus arteriosus was located on the right side of the trachea; (F) The CPVC connected to the superior vena cava. (AAO = ascending aorta, AO = aorta, CPVC = common pulmonary veins chamber, DA = ductus arteriosus, DAO = descending aorta, LCCA = left common carotid artery, LIA = left innominate artery, LLU = left lung, LPA = left pulmonary artery, LSA = left subclavian artery, MPA = main pulmonary artery, RCCA = right common carotid artery, RLU = right lung, RSA = right subclavian artery, RV = right ventricle, T = trachea).

No other extra-cardiac findings or malformation of fetal systems were detected. One week later the woman requested an abortion and consented to an autopsy. The autopsy supported the echocardiographic findings (Fig. 3A–F). Autopsy also revealed that the fetus had no spleen and a bilateral trilobed lung, which were difficult to visualize on ultrasound.

## 3 Discussion

RAI is a rare congenital disease that was first described by Pohlius in 1940. In 1955, Ivemark inferred that RAI might be caused by a genetic mutation and autosomal recessive inheritance, also known as Ivemark syndrome.^[5]^ Anatomically, RAI is characterized by the absence of a spleen or the presence of a rudimentary spleen; visceral malformation or heterotopia of the liver and stomach; anomalies of multiple atrioventricular structures; and valvular malformation.^[5]^ RAI is associated with substantial morbidity and mortality. Most patients with RAI die within first year of life due to cardiovascular compromise;^[6]^ however, outcomes following surgery for RAI are improving. A retrospective review of patients with RAI who underwent surgical repair of their cardiac defects showed that 5-year survival had increased from 53.8% in 1997 to 2003 to 81.7% in 2004 to 2010.^[7]^ Further, a review of the medical records of adults with congenital heart disease and heterotaxy syndrome from 1964 to 2018 revealed that improvements in early diagnosis, neonatal management, and technical capabilities available to healthcare providers had enabled many patients to accomplish important life milestones such as higher education, full-time employment, marriage, and becoming parents.^[8]^

Accurate diagnosis of RAI is essential for clinical and parent decision making. Sonographic imaging of RAI is so complex that conventional 2D-Doppler imaging may lead to a misdiagnosis. For example, the spatial relationships between blood vessels may be overlooked on 2D-Doppler imaging. Alternatively, STIC-HD live flow can vividly display the spatial relationships between blood vessels in a stereoscopic mode.

However, STIC-HD live flow cannot distinguish lung lobes. The fetal lung has no respiratory function; therefore, it often appears as an isoechoic mass on prenatal ultrasound. In the present case, a bilateral trilobed lung was seen on autopsy. Other findings on prenatal ultrasound were confirmed via autopsy, including the atrioventricular septal defect, supra-cardiac TAPVC, a double outlet right ventricle, a RAA with mirror image branching and aortic arch dysplasia.

In summary, RAI presenting with an atrioventricular septal defect, a persistent left superior vena cava, supra-cardiac TAPVC, a double outlet right ventricle, a right ductus arteriosus, a right aortic arch with mirror image branching and aortic arch dysplasia is a very rare combination of fetal defects. To the best of our knowledge, the present case is the first report describing such complex cardiac comorbidities in a fetus with RAI. Effective approaches to prenatal detection of the anatomic factors associated with RAI are required to facilitate counseling of expectant parents about prognosis and inform clinical decision making in the neonatal period and during follow up into adulthood. We described the complexity of the heart defects in this fetus using STIC-HD live flow and autopsy. Findings will raise awareness about the heterogeneity of the cardiac abnormalities associated with RAI and the need for new therapeutic approaches.

## Author contributions

LY and LZ participated in the design of the study and collected the clinical data. LY processed the data. LY performed the statistical analysis. LZ and LC helped to interpret the results. LY drafted the manuscript. All authors read and approved the final manuscript.

**Data curation:** Linhua Yang, Liuying Zhou, Lin Chen.

**Formal analysis:** Linhua Yang.

**Project administration:** Linhua Yang, Liuying Zhou.

**Software:** Linhua Yang, Lin Chen.

**Visualization:** Liuying Zhou.

**Writing – original draft:** Linhua Yang.
